# Fitness Costs and the Rapid Spread of *kelch13*-C580Y Substitutions Conferring Artemisinin Resistance

**DOI:** 10.1128/AAC.00605-18

**Published:** 2018-08-27

**Authors:** Shalini Nair, Xue Li, Grace A. Arya, Marina McDew-White, Marco Ferrari, François Nosten, Tim J. C. Anderson

**Affiliations:** a Texas Biomedical Research Institute, San Antonio, Texas, USA; b Department of Biosciences, University of Milan, Milan, Italy; c Shoklo Malaria Research Unit, Mahidol-Oxford Tropical Medicine Research Unit, Faculty of Tropical Medicine, Mahidol University, Mae Sot, Thailand; d Centre for Tropical Medicine and Global Health, University of Oxford, Oxford, United Kingdom

**Keywords:** CRISPR/Cas9, amplicon sequencing, fitness costs, selection coefficient

## Abstract

Fitness costs are key determinants of whether drug resistance alleles establish and how fast they spread within populations. More than 125 different *kelch13* alleles, each containing a different amino acid substitution, have arisen in Southeast Asian malaria parasite (Plasmodium falciparum) populations under artemisinin selection over the past 15 years in a dramatic example of a soft selective event. However, just one of these alleles (C580Y) is now outcompeting other alleles in multiple different countries and is spreading toward fixation. Here we examine the fitness consequences of C580Y, relative to another less successful *kelch13* mutation (R561H), to try to explain the distinctive dynamics of C580Y. We hypothesized that C580Y will show lower fitness costs than other *kelch13* substitutions in the absence of artemisinin treatment. We used CRISPR/Cas9 methods to introduce single mutations (C580Y or R561H) or synonymous control edits into a wild-type parasite isolated on the Thailand-Myanmar border, conducted replicated head-to-head competition assays, and determined the outcome of competition using deep sequencing of *kelch13* amplicons. Contrary to our predictions, these experiments reveal that C580Y carries higher fitness costs (*s* [selection coefficient] = 0.15 ± 0.008 [1 standard error {SE}]) than R561H (*s* = 0.084 ± 0.005). Furthermore, R561H outcompetes C580Y in direct competition (*s* = 0.065 ± 0.004). We conclude that fitness costs of C580Y in isolation are unlikely to explain the rapid spread of this substitution.

## Abstract (precis)

Fitness costs are key determinants of whether drug resistance alleles establish and how fast they spread within populations. More than 125 different *kelch13* alleles, each containing a different amino acid substitution, have arisen in Southeast Asian malaria parasite (Plasmodium falciparum) populations under artemisinin selection over the past 15 years in a dramatic example of a soft selective event.

## INTRODUCTION

Two opposing forces determine whether resistance alleles establish within populations and how rapidly they spread. Resistance alleles that confer the greatest ability to survive drug treatment and be transmitted to new hosts will be most likely to escape loss by genetic drift and will also increase in frequency within populations. However, resistance alleles typically disrupt parasite metabolic pathways and so are also expected to carry a fitness cost in the absence of drug treatment (1, 2). Resistance alleles conferring low fitness costs are expected to outcompete and replace alleles with high fitness costs. Fitness costs of resistance alleles may be high in nature, so they are important to quantify. When chloroquine was withdrawn from Malawi, resistance alleles plummeted from an 85% frequency in 1982 to 0% in 2001 (3). This is equivalent to a selection coefficient (*s*) of 0.07 to 0.13 per Plasmodium generation for sensitive alleles relative to the resistance allele at the chloroquine resistance transporter locus (4).

In Southeast Asia, at least 125 independent nonsynonymous substitutions in the *kelch13* locus conferring artemisinin (ART) resistance (ART-R) have arisen in malaria (Plasmodium falciparum) populations treated with artemisinin-based combination therapies (5), in a dramatic example of a soft selective sweep. However, just one of these mutations (C580Y) is currently replacing other ART-R substitutions both at the Thailand-Myanmar border and in Cambodia, Vietnam, and southern Lao (6–8). C580Y has independent origins in these two regions of Southeast Asia (6, 9, 10). The rapid spread of independently derived C580Y alleles in two weakly connected malaria parasite populations is unlikely to be explained by chance. A practical concern is that this successful ART-R allele may eventually spread westwards and establish in sub-Saharan Africa, following the path of chloroquine, pyrimethamine, and sulfadoxine resistance (11–13).

What makes C580Y so successful compared to other *kelch13* substitutions? There are several possible explanations. (i) C580Y may show greater survival than other ART-R alleles following artemisinin treatment. Two lines of evidence argue against this. First, the rate of parasite clearance following artemisinin treatment (measured by the clearance half-life, *T*_1/2_*P*) is now available for thousands of P. falciparum-infected patients. C580Y (*T*_1/2_*P* = 6.35 ± 0.11 h[1 standard error {SE }]) shows an intermediate *T*_1/2_*P* and is comparable to many other *kelch13* substitutions (6). Second, patients infected with parasites bearing C580Y showed risks of treatment failure comparable to those of patients infected with parasites bearing other *kelch13* alleles (14). (ii) Parasites bearing C580Y may produce more gametocytes than those bearing other ART-R alleles or show higher fitness in the liver stage or during mosquito development. In the largest treatment failure study to date, there was no association between infections bearing ART-R *kelch13* mutations and gametocyte carriage (14).

Here we examine a third hypothesis, that C580Y is associated with lower fitness costs than other ART-R substitutions in the absence of drug pressure. This would allow parasites bearing C580Y to outcompete parasites carrying other *kelch13* substitutions within mixed infections and to reach high parasite densities needed for transmission more rapidly than parasites carrying other alleles in monoclonal infections. We examine this hypothesis by introducing C580Y or another *kelch13* substitution (R561H) onto the same wild-type genetic background and then directly quantifying fitness costs during *in vitro* parasite culture using replicated head-to-head competition experiments.

## RESULTS

**FIG 1:**
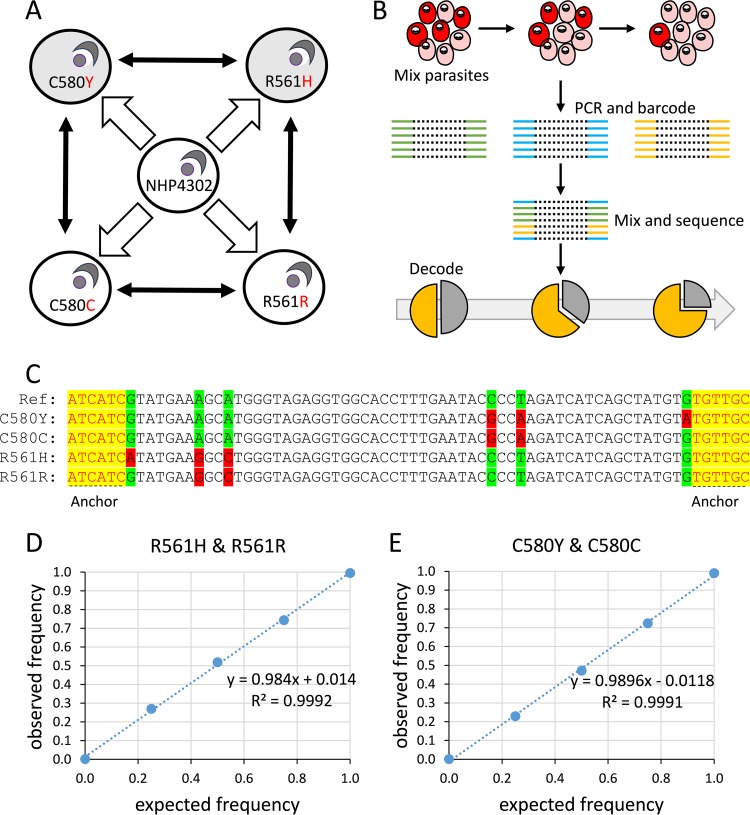
Study design. (A) We generated 4 different edited parasites from a single ART-S parasite clone (NHP4302). Two of the edited parasites (gray shading) have edited bases conferring ART-R, while two control edits (white shading) have synonymous shield mutations only. The amino acids present in edited parasites are shown in red. The black two-headed arrows show the four head-to-head competition experiments conducted. (B) Overview of amplicon sequencing for parasite competition experiments. (C) Sequences used for assigning reads to different competing parasite lines. Raw reads were chopped and aligned by using anchor sequences at each end of the CRISPR-edited region. We then used the mutations present to determine the frequencies of the competing parasites. (D and E) Observed and expected allele frequencies of NHP4302^R561H^ (D) and NHP4302^C580Y^ (E) from artificial mixtures.

We compared fitnesses of blood-stage parasites for two different mutations, C580Y and R561H. Parasites with R561H show very low clearance rates (*T*_1/2_*P* = 6.99 h ± 0.21 h [1 SE]) (6). The frequency of this substitution peaked at 10% in 2012 and has subsequently declined, while the frequency of C580Y has increased, rising from 4% in 2010 to 65% in 2014 (6). We used CRISPR/Cas9 to generate 4 edited parasite clones derived from NHP4302 (*T*_1/2_*P* = 1.98 h), a cloned *kelch13* wild-type parasite (Fig. 1A). NHP4302^C580Y^ has a nonsynonymous mutation encoding C580Y as well as two synonymous shield mutations to prevent the repeated cutting of target sites in edited parasites by Cas9. NHP4302^R561H^ has the nonsynonymous mutation encoding R561H and two synonymous shield mutations. We also constructed control edits: NHP4302^C580C^ and NHP4302^R561R^ have two synonymous shield mutations but no amino acid changes. These parasites serve as controls for off-target edits, or mutations occurring during the recovery of edited parasites, and are the artemisinin-sensitive (ART-S) *kelch13* clones used for growth comparisons.

We conducted 4 head-to-head competition experiments (6 replicates of each) to measure fitness consequences of the introduced mutations (Fig. 1A). These experiments included the following comparisons: ART-R NHP4302^C580Y^ versus ART-S NHP4302^C580C^ parasites, ART-R NHP4302^R561H^ versus ART-S NHP4302^R561R^ parasites, direct comparison of the two ART-R parasites (NHP4302^C580Y^ versus NHP4302^R561H^), and direct comparison of the two ART-S control parasites (NHP4302^C580C^ versus NHP4302^R561R^). We used deep sequencing of a 249-bp fragment of *kelch13* (positions 1725146 to 1725395) on a MiSeq 2.6.2.1 instrument (Illumina) to determine frequencies of the two competing parasite lines in each experiment (Fig. 1B; see also Data Set S1 in the supplemental material). We harvested a total of 12.3 million paired-end 250-bp reads from this MiSeq run.

**FIG 2:**
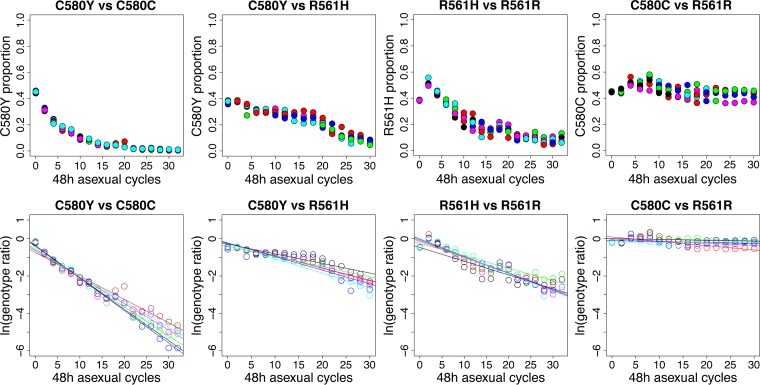
Head-to-head competition experiments. (Top) Trajectory of competing parasites observed in six replicate experiments (each color represents one replicate). (Bottom) Natural log of the parasite ratio against time between competing parasites. The slope for the least-squares fit provides an estimate of the selection coefficient (*s*).

We deconvoluted sequence files to identify the time point, replicate, and experiment and used custom scripts (Fig. 1C and Text S1) to determine frequencies of competing parasites in each mixture (or experiment). We obtained 24,878 ± 394 (±1 SE) sequence reads from the each of the aliquots in these experiments (Data Set 2). A total of 0.87 to 5.42% of sequences could not be scored for the bases distinguishing the competing parasites and were excluded. We sequenced artificial mixtures containing NHP4302^C580Y^ and NHP4302^C580C^ or NHP4302^R561H^ and NHP4302^R561R^ in proportions of 0:1, 0.25:0.75, 0.5:0.5, 0.75:0.25, and 1:0 to evaluate the accuracy of allele frequency estimates: *r*^2^ values of 0.9992 and 0.9991 between observed and expected frequencies demonstrate the accuracy of this approach (Fig. 1D and E and Data Set 2). We plotted proportions of the competing parasites across time in our head-to-head competition experiments and used the raw data to determine selection coefficients for each replicate and for all replicates combined (Fig. 2).

**FIG 3:**
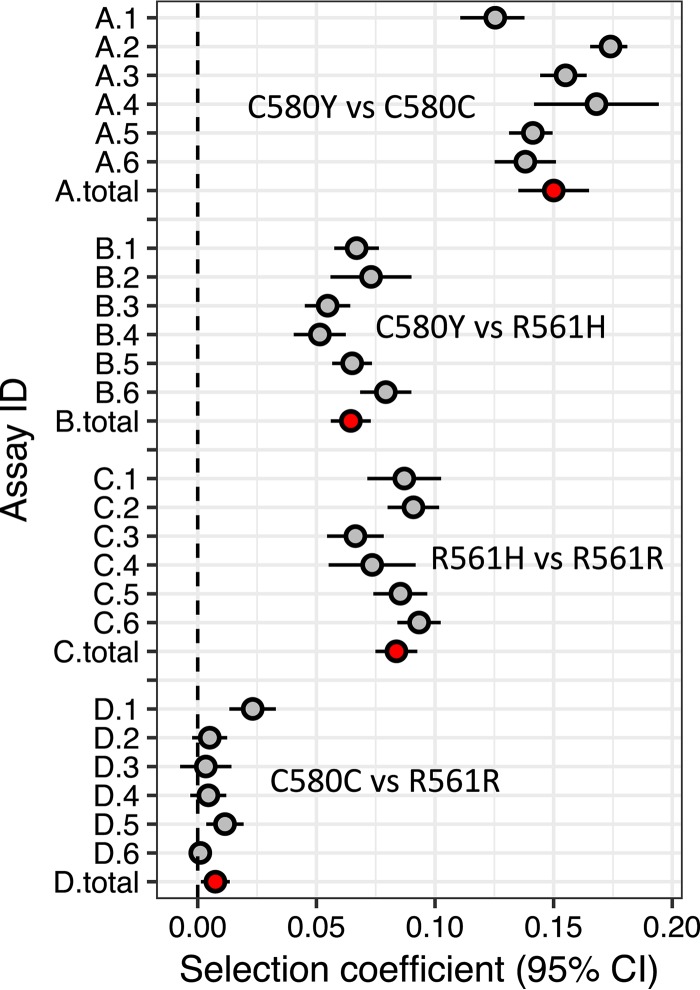
Quantifying the outcomes of head-to-head competition. The plot shows selection coefficients (*s*) with 95% confidence intervals (CI) from 4 sets of competition experiments. Six replicate competition experiments were conducted for each set: gray points show *s* for each replicate, while red points show meta-analysis results for each experimental comparison. The “winning” parasite is shown on the right for each comparison.

Plots of natural log (parasite ratio) show a good fit to linear expectations (Table 1), and replicates within experiments are extremely consistent (Fig. 2 and 3). Both experiments comparing ART-S controls with ART-R parasites reveal substantial fitness costs of *kelch13* mutations (Fig. 2 and 3). Fitness costs were higher for NHP4302^C580Y^ (*s* = 0.15 ± 0.008 [1 SE]) than for NHP4302^R561H^ (*s* = 0.084 ± 0.005 [1 SE]) in competition experiments against ART-S controls. Consistent with this, NHP4302^R561H^ outcompeted NHP4302^C580Y^ in direct competition (*s* = 0.065 ± 0.004 [1 SE]) (Fig. 3). These experiments show good internal consistency: the fitness difference observed for direct competition (*s* = 0.065) between ART-R parasites should equal the fitness difference measured for competition between the ART-R mutants and ART-S controls (0.150 − 0.084 = 0.066).

**TABLE 1:** Calculation and summary of selection coefficients for all competition experiments^*a*^

Strains in culture	Assay	Mean *s* ± SE	*R*^2^	No. of time points	*P*
C580Y × C580C	A.1	0.125 ± 0.008	0.936	17	2.275E−10
	A.2	0.174 ± 0.005	0.988	17	6.664E−16
	A.3	0.155 ± 0.006	0.977	17	1.001E−13
	A.4	0.168 ± 0.014	0.969	7	5.998E−05
	A.5	0.141 ± 0.006	0.976	17	1.523E−13
	A.6	0.138 ± 0.007	0.965	15	8.140E−11
	A.total	0.150 ± 0.008	NA	90	1.535E−86
C580Y × R561H	B.1	0.067 ± 0.005	0.918	16	5.670E−09
	B.2	0.073 ± 0.010	0.801	16	2.873E−06
	B.3	0.055 ± 0.005	0.913	12	1.258E−06
	B.4	0.051 ± 0.006	0.829	16	9.906E−07
	B.5	0.065 ± 0.005	0.930	16	1.866E−09
	B.6	0.079 ± 0.006	0.945	12	1.226E−07
	B.total	0.065 ± 0.004	NA	88	1.065E−51
R561H × R561R	C.1	0.087 ± 0.009	0.882	15	2.120E−07
	C.2	0.091 ± 0.006	0.939	16	6.785E−10
	C.3	0.066 ± 0.007	0.863	17	7.408E−08
	C.4	0.073 ± 0.010	0.779	16	5.935E−06
	C.5	0.085 ± 0.006	0.931	15	6.410E−09
	C.6	0.093 ± 0.005	0.955	17	1.635E−11
	C.total	0.084 ± 0.005	NA	96	8.392E−79
C580C × R561R	D.1	0.023 ± 0.006	0.549	16	0.001
	D.2	0.005 ± 0.004	0.092	16	0.254
	D.3	0.003 ± 0.006	0.029	16	0.599
	D.4	0.004 ± 0.004	0.082	14	0.320
	D.5	0.011 ± 0.004	0.356	14	0.024
	D.6	0.001 ± 0.002	0.013	16	0.676
	D.total	0.007 ± 0.003	NA	92	0.017

a Selection coefficients (*s*) (means ± standard errors) were measured as described in the legend of Fig. 2. For each combination, we conducted six independent competition assays. The final three columns report the correlation coefficient (*R*^2^), the number of time points at which genotype frequencies were measured, and the statistical significance (*P*) for a test of the null hypothesis that the slope is not different from zero. The winning parasite clone is shown on the right for each comparison. Time points with failed sequencing were removed from the analysis. NA, not applicable.

We observed 152 mutations, including 31 single nucleotide polymorphisms (SNPs) and 122 indels, across the genome, in addition to the specific edits introduced by CRISPR/Cas9 (Data Set S3). These numbers are consistent with observations from previous Plasmodium CRISPR/Cas9 experiments in which 115 and 126 indels were identified in two edited clones sequenced (15). We do not expect to see off-target effects given Plasmodium's inefficient end-joining pathway (16); consistent with this, none of the mutations are found near protospacer adjacent motif (PAM) sites. These mutations accumulate during asexual culture. The NHP4302 clone was cultured for a cumulative total of 46 days after dilution cloning and prior to editing, followed by 18 to 20 days after selection to grow out edited cells. Hence, mutations accumulated for 64 to 66 days and were then fixed when we cloned edited parasites from a single cell. Reassuringly, the two ART-S parasites edited with shield mutations show comparable fitness, suggesting that the mutations that accumulated during the editing process do not have an impact.

We further checked genetic loci that might influence the fitness of artemisinin-resistant parasites. Miotto et al. (17) suggested that mutations at the genes *pffd* (ferredoxin) (D193Y), *pfarps10* (apicoplast ribosomal protein S10) (V127M), *pfmdr2* (multidrug resistance protein 2) (T484I), and *pfcrt* (chloroquine resistance transporter) (N326S) are markers of the genetic background for *kelch13* mutations arising in Southeast Asia. We scanned these four loci and found two mutations, *pfcrt*-N326S and *pfarps*10-V127M, in the initial NHP4302 parasite and the four edited parasites.

## DISCUSSION

These results demonstrate high fitness costs (*s* = 0.084 to 0.150) associated with two ART-R *kelch13* mutations in Southeast Asia. These costs were measured by changing single amino acids on the same parasite genetic background. Furthermore, to maximize relevance to the field, we used a recently isolated parasite clone from the Thailand-Myanmar border for manipulation rather than a laboratory-adapted parasite clone. We also used synonymous edits to generate sensitive parasites and control for off-target effects or mutations accumulating during CRISPR/Cas9 editing. The strong deleterious effects of these ART-R mutations are consistent with the evolutionary conservation across Plasmodium species (8, 18): without artemisinin pressure, there is strong purifying selection preventing amino acid changes in *kelch13*.

ART-R alleles, in particular C580Y, are spreading in Southeast Asia under ART treatment. Consistent with this, ART-R parasites outcompete the ART-S parasites from which they were derived in laboratory competition under artemisinin pressure (19). We have estimated that selection coefficients driving the spread of *kelch13* mutations on the Thailand-Burma border are between 0.085 and 0.12 (depending on assumptions about the duration of infection in the human host), based on allele frequency changes over time. For C580Y, *s* driving spread is much higher (*s* = 0.2). Note that these estimates of *s* are estimated for each Plasmodium generation, spanning both parasite development in the mosquito and asexual growth in the human host, while our experimental measures of *s* are calculated for each 48-h asexual replication cycle. The extremely high costs of resistance in the absence of drug pressure make the rapid spread of C580Y all the more remarkable. In this paper, we do not address the phenotypic changes that underlie reduced fitness. One possibility is that changes in the length of the asexual life cycle (20) may contribute to the fitness differences observed.

Contrary to our predictions, the successful C580Y mutation showed much higher fitness costs than R561H, which is diminishing in frequency on the Thailand-Myanmar border. This result suggests that a decreased fitness cost of the C580Y substitution in isolation is unlikely to explain its success. Our results receive some support from an independent project. Tirrell et al. (A. R. Tirrell, L. A. Checkley, K. M. Vendrely, M. McDew-White, A. M. Vaughan, F. H. Nosten, T. J. C. Anderson, and M. T. Ferdig, unpublished data) conducted pairwise head-to-head competitions between 3 ART-S and 5 ART-R clones from the Thailand-Myanmar border. C580Y ranked below two other ART-R clones (E252Q and G538V) in relative fitness. This experiment competed clones of field-isolated parasites that differed both at *kelch13* and at perhaps thousands of SNPs throughout the genome; nevertheless, their results are consistent with ours in providing no indication that fitness costs incurred by isolates carrying the C580Y mutation are lower than those of other ART-R alleles.

So what explains the success of C580Y? Epistatic interactions with other variants in the parasite genome provide the most persuasive explanation for the success of C580Y. Our experiments examined the impact of the C580Y substitution in isolation on parasite fitness. Several lines of evidence suggest that variants elsewhere in the genome may be needed to compensate for the deleterious fitness effects of *kelch13* mutations. Strong associations between ART-R and regions other than *kelch13* have been identified in association studies (17, 21, 22), with regions on chromosome 14 showing consistent associations among studies. Miotto et al. (17) suggested that a particular genetic background, with specific variants at 4 loci, are permissive for the evolution of resistance. The ART-S parasite used in this work has 2/4 of these variants (*pfcrt*-N326S and *pfarps*10-V127M). Cerqueira et al. (23) noted that the frequencies of some P. falciparum SNPs are increased on the Thai-Myanmar border at rates similar to those of *kelch13*, suggesting the involvement of other loci. Those authors show strong linkage disequilibrium between *kelch13* mutations and another locus encoding a kelch-like protein on chromosome 10, providing further evidence that genetic background is important.

Perhaps the most direct evidence for epistasis and compensatory mutations comes from recent work examining fitness costs using recently isolated Cambodian parasites (24). Those researchers observed no change in fitness when C580Y was introduced into recently isolated Cambodian parasites but measured high fitness costs (*s* = 0.11) when C580Y was introduced into a laboratory parasite line (VI/S) isolated prior to the use of artemisinin. The absence of fitness costs associated with C580Y in the recent Cambodian isolates strongly suggests the involvement of compensatory loci. The study by Straimer et al. (24) used recently isolated Cambodian parasites, while this study used a recently isolated wild-type parasite from the Thailand-Myanmar border, where C580Y has evolved independently. While sample sizes are small, these studies hint that background mutations may differ in these two malaria populations.

Parasites bearing *kelch13*-C580Y together with duplications of a chromosome 14 region containing *Plasmepsin2* associated with piperaquine resistance (25, 26) have recently spread from Western Cambodia into Southern Laos and Northeastern Thailand and Vietnam (7, 10, 27); this has probably fueled the recent rapid spread of C580Y across these countries. However, linkage disequilibrium between these two drug resistance alleles does not help to explain why C580Y outcompeted other ART-S alleles prior to the emergence of piperaquine resistance.

The high fitness costs of *kelch13* mutations (and particularly C580Y) may present strong barriers preventing the establishment and spread of *kelch13* substitutions arising *de novo* in African parasite populations. In particular, such barriers may be elevated in high-transmission regions where (i) emerging ART-R mutations must compete with coinfecting wild-type parasites; (ii) asymptomatic infections are common, so the proportion of the parasite population exposed to artemisinin selection is much lower; and (iii) high levels of recombination disrupt associations with compensatory alleles elsewhere in the genome (28). The potential for optimized Southeast Asian parasite genotypes containing *kelch13* mutations coupled with compensatory changes to establish and spread in sub-Saharan malaria parasite populations should be taken seriously (23).

Genetic crosses are now possible in Plasmodium by using humanized mice (29). Crosses between ART-R and ART-S parasites will allow shuffling of ART-R *kelch13* mutations and epistatically acting genes elsewhere in the genome. This should provide a powerful approach for identifying compensatory mutations that restore parasite fitness associated with ART-R substitutions in *kelch13*.

The deep amplicon sequencing method that we used here provides a robust alternative to pyrosequencing (24) for quantifying the outcomes of head-to-head competition experiments that is easily scalable to measure growth rates in large numbers of comparisons. Similar amplicon-based approaches are now widely used for characterizing the composition of natural malaria infections (30, 31). Amplicon sequencing has particular advantages over pyrosequencing for assessing the outcomes of competition experiments because the readout is digital, and we can determine frequencies of haplotypes containing several SNPs on the same amplicon and resolve the outcomes of competition experiments involving multiple competing parasites.

## MATERIALS AND METHODS

### Parasite and culture.

We used a parasite bearing wild-type *kelch13* isolated from a patient visiting the Wang Pha clinic run by the Shoklo Malaria Research Unit (SMRU) in 2008 (NHP4302; *T*_1/2_*P* = 1.98 h). This parasite isolate was grown in the laboratory and cloned by limiting dilution, and a single parasite clone was used for these experiments. We cultured all asexual blood-stage parasites in standard Plasmodium culture medium (RPMI 1640 supplemented with 2 mM l-glutamine, 25 mM HEPES, and 50 μg/liter gentamicin) at 2% hematocrit and maintained them at 37°C with 5% O_2_, 5% CO_2_, and 90% N_2_.

### CRISPR/Cas9 gene editing.

We conducted gene edits using plasmids engineered as described previously by Ghorbal et al. (15) and used infusion cloning approaches to introduce donor sequences and guide sequences (see Data Set S1 in the supplemental material), followed by Sanger sequencing to check constructs. We transfected the plasmids into NHP4302 by electroporation and selected successful transfections by applying drug pressure for 6 days. We used 5 nM WR99210 (Jacobus Pharmaceuticals, Princeton, NJ) and 1.5 μM DSM1 (BEI Resources, Manassas, VA) as described previously by Ghorbal et al. (15) and Straimer et al. (32). After 18 to 20 days, we recovered parasites and tested for modifications using restriction DNA digests of bulk cultures, using BfaI (CTAG) for C580Y/C580C and StuI (AGGCCT) for R561H/R561R. We further cloned the parasite cultures with positive edits by limiting dilution and confirmed the edits in each clone by Sanger sequencing. We selected one clone from each of the 4 editing experiments for further work. We were concerned about off-target edits, plasmid integration, and mutations accrued during editing, so we genome sequenced all 4 parasite clones.

### Whole-genome sequencing and data processing.

We genome sequenced the four CRISPR-edited parasite lines and the original parasite isolate, NHP4302, to assess the possibility of off-target edits, plasmid integration, and mutations accrued during editing. Genomic DNA was extracted and purified by using a Qiagen DNA minikit. We constructed next-generation sequencing libraries according to the Kapa HyperPlus kit protocol. All libraries were sequenced to an average coverage of >100× by using an Illumina HiSeq 2500 sequencer (21). We used the P. falciparum-adapted CRISPR plasmids pUF1_Cas9_InFusion and pL_6_eGFP_1 in this study (15). To check if any plasmid integrated to the parasite genome, we generated a dummy genome by merging sequences of the P. falciparum 3D7 genome (http://plasmodb.org/common/downloads/release-32/Pfalciparum3D7/) and the two plasmid sequences. Paired-end 100-bp whole-genome sequencing reads for each edit were individually mapped against this dummy genome by using the alignment algorithm BWA mem (http://bio-bwa.sourceforge.net/) under the default parameters. The resulting pileup files were further converted to SAM format, sorted to BAM format, and deduplicated by using picard tools v2.0.1 (http://broadinstitute.github.io/picard/).

We visualized reads mapping to plasmids using the Integrative Genomics Viewer (IGV) and compared parasites before and after CRISPR editing. We see no evidence that plasmid integration occurred during CRISPR/Cas9 editing.

We called SNPs and small indels using Genome Analysis Toolkit (GATK) v3.7 (https://software.broadinstitute.org/gatk/) according to best-practice recommendations but with minor adaptations for P. falciparum. We recalibrated base quality scores based on a set of verified known variants (33). We first called variants independently for each parasite line using HaplotypeCaller and then merged them by using GenotypeGVCFs, according to default parameters but with –sample_ploidy 1. To avoid false positives, we applied quality criteria (QualByDepth of >2.0, FisherStrand of <60, RMSMappingQuality of >40.0, MappingQualityRankSumTest >−12.5, StrandOddsRatio of <4.0, and ReadPosRankSumTest of >−8.0) to filter the original GATK genotypes. We excluded variants from variable regions (subtelomeric repeats, hypervariable regions, and centromeres) of the P. falciparum genome (33). As the Cas9 protein is transiently expressed (15) during CRISPR and all the parasite lines were originally obtained from single-cell cloning of CRISPR-edited parasites, any SNPs or indels from off-target activity should appear in 100% of the cloned population. At each candidate nucleotide position, we required >90% of the reads to indicate the same nucleotide, with a minimum of 4 paired-end reads being required. The P. falciparum genome is extremely AT rich and highly repetitive and tends to exhibit a high level of mutability (34); candidate mutations located inside the tandem repeat array were not considered.

Sequences 23 bp upstream to 23 bp downstream from the position of each mutation were compared with the guide sequence, and we calculated the probability (statistical significance) that these sequences are targets of the guide sequence using the formula described previously by Cho et al. (35). We visually checked edited regions in *kelch13* using IGV to verify the edited mutations.

### Amplicon sequencing for parasite competition experiments.

We conducted head-to-head competition experiments to measure fitness consequences of the introduced mutations. We synchronized the *kelch13*-edited parasites to 80% late schizonts using MACS purification columns (Miltenyi Biotec) and adjusted the cultures to 0.1% parasitemia. We cultured the parasites overnight until early ring stages and mixed different edits in a 1:1 ratio to set up competition growth assays.

We conducted all experiments in 25-ml flasks under standard malaria parasite culture conditions. To minimize experimental variation, we used the same red blood cell donor for all experiment. We checked the parasites every 2 days and split cultures to reduce parasitemia to 0.1%, if parasitemia was >1%. We maintained the culture for 60 days and removed 80 μl packed red blood cells every 4 days to monitor the outcome of competition.

We amplified a 249-bp region located in *kelch13* that including all desired and shield mutations by Illumina-adapted primers incorporating 12-bp barcodes (36, 37) (see Data Set 1 in the supplemental material). We cleaned PCR products with AMPure beads and quantified products using a standard PicoGreen assay that can be read on a fluorescent plate reader. Equimolar amounts of samples were pooled, diluted to 2 nM, and quantified with the Kapa library quantification kit. We further diluted libraries to 8 pM and mixed them with 8 pM of PhiX before they were loaded onto the MiSeq instrument.

We pooled and sequenced a total of 371 purified PCR products in a single MiSeq run. We used PCR products from artificial mixture of parasites as a control. The 40% PhiX library was spiked into the total pooled PCR product to balance the extremely low diversity of the amplicons sequenced. Illumina standard primers were mixed with the custom sequencing primers and loaded according to the manufacturer's instructions.

We used custom scripts to determine frequencies of competing parasites in each sample (Text S1 and Data Set S2). Raw reads with >20% bases with a quality score of <20 were removed from further analysis. The quality-filtered reads were trimmed to 70 bp using two 6-bp anchors at each end of the CRISPR-edited region (Fig. 1C). We scored 6 variable bases to determine the clone of origin of the amplicon sequences. We plotted the natural log of the parasite ratio (frequency of clone A/frequency of clone B) against time (measured in 48-h parasite asexual cycles) and fitted a linear model for six independent competition assays with each parasite combination (Fig. 2 and Data Set S2). The slope provides a measure of the selection coefficient (*s*), a measure of the proportional growth differential between the two competing parasites at each asexual generation (38). Slopes from six replicate competition assays were combined by using the R package metafor with a random-effects analysis (39).

### Data availability.

Raw sequencing data have been submitted to the NABI Sequence Read Archive (SRA) (https://www.ncbi.nlm.nih.gov/sra) under project accession number PRJNA435422.

## Supplementary Material

Supplemental file 1

Supplemental file 2
