# Supplementary material for: Fitness Costs and the Rapid Spread of kelch13-C580Y Substitutions Conferring Artemisinin Resistance
Source: Antimicrob Agents Chemother. 2018 Aug 27;62(9):e00605-18. doi: 10.1128/AAC.00605-18 (PMC6125530; doi:10.1128/AAC.00605-18)
Supplement: Supplemental file 1 [file zac009187413s1.pdf]

Supplementary file 1. Scripts to determine frequencies of competing parasites.

```
#!/bin/bash

## (1) demultiplex samples

ulimit -n 4000

bcl2fastq -o ./out

unzip *


## (2) reverse and complement read2 if pair-end sequencing using FASTX-Toolkit (http://hannonlab.cshl.edu/fastx_toolkit/) from Hannon lab.

for file in $(ls)

do

fastx_reverse_complement -i $file -o $file-reco -Q 33

done


## (3) merge two reads

for file in $(ls)

do

cat $file $file-reco > mergeR1R2/$file-merge.fastq

done


## (4) separate reads. Perl script extract.pl used here was attached in the end.

for file in $(ls mergeR1R2/)

do

perl extract.pl -i mergeR1R2/$file -o Anchored/$file -b ATCATC[ATGC]{58}TGTTGC -l 70

perl extract.pl -i mergeR1R2/$file -o Ref/$file -b ATCATCG[ATGC]{6}A[ATGC]{2}A[ATGC]{26}C[ATGC]{2}T[ATGC]{16}GTGTTGC -l 70

perl extract.pl -i mergeR1R2/$file -o 580Mut/$file -b ATCATCG[ATGC]{6}A[ATGC]{2}A[ATGC]{26}G[ATGC]{2}A[ATGC]{16}ATGTTGC -l 70
```

```
perl extract.pl -i mergeR1R2/$file -o 580Wt/$file -b
ATCATCG[ATGC]{6}A[ATGC]{2}A[ATGC]{26}G[ATGC]{2}A[ATGC]{16}GTGTTGC -l 70

perl extract.pl -i mergeR1R2/$file -o 561Mut/$file -b
ATCATCA[ATGC]{6}G[ATGC]{2}C[ATGC]{26}C[ATGC]{2}T[ATGC]{16}GTGTTGC -l 70

perl extract.pl -i mergeR1R2/$file -o 561Wt/$file -b
ATCATCG[ATGC]{6}G[ATGC]{2}C[ATGC]{26}C[ATGC]{2}T[ATGC]{16}GTGTTGC -l 70

done


## (5) count reads

for file in $(ls mergeR1R2/)

do

awk '{s++}END{print s/4}' mergeR1R2/$file >> Total.txt

awk '{s++}END{print s/2}' -l Anchored/$file >> Anchored.txt

awk '{s++}END{print s/2}' -l 580Mut/$file >> 580Mut.txt

awk '{s++}END{print s/2}' -l 580Wt/$file >> 580Wt.txt

awk '{s++}END{print s/2}' -l 561Mut/$file >> 561Mut.txt

awk '{s++}END{print s/2}' -l 561Wt/$file >> 561Wt.txt

done
```

```
##!/usr/bin/perl -w
### Extract the target site ###
my $l=0;
my $Q1=20;
my $Q2=0.2;
parse_command_line();
open FIG,    "<$input";
open OUT,    ">$output";
$cnt=0;
foreach $word(<FIG>){
        chomp($word=$word);
        @file=split(/\s+/,$word);
        $cnt=$cnt+1;
        if($cnt%4==1){$name=substr($word,1);}
        elsif($cnt%4==2){$read=$word;}
        elsif($cnt%4==0){$qual=$word;
            $len=length $read;
            $qual_sum=0;
           # Quality filter
            for($i=0;$i<$len;$i++){
                        $tt=substr($qual,0,$i);
                        $tt=ord($tt)-33;
                        if($tt<$Q1){$qual_sum++;}
            }
                                        # Extract the target site
            if($qual_sum>=$len*$Q2){;}
            else{
                $tag=0;
                if($read=~/$base/){
```

```
                    while($read=~/$base/g ){
                    $end=pos($read);
                    $ss=substr($read,$end-$l,$l);
                    $tag++;
                    }
                print OUT ">$name\n$ss\n";
                }
            }
        }
}
sub parse_command_line {
    while (@ARGV) {
            $_ = shift @ARGV;
                if      ($_ =~ /^-i$/)  { $input   = shift  @ARGV; }
        elsif    ($_ =~ /^-o$/)  { $output  = shift  @ARGV; }
        elsif    ($_ =~ /^-b$/)  { $base    = shift  @ARGV; }
        elsif    ($_ =~ /^-l$/)  { $l       = shift  @ARGV; }
        elsif    ($_ =~ /^-Q1$/) { $Q1      = shift  @ARGV; }
        elsif    ($_ =~ /^-Q2$/) { $Q2      = shift  @ARGV; }
        else {
                  print STDERR "Unknown command line option: '$_'\n";
                  usage();
                }
    }
}
sub usage {
    print STDERR <<EOQ;
    perl reads_filter.pl -i input  -o output -b -Q1  -Q2 [-h]
    i    :input file
```

```
    o    :outputfile
    b    :target site : ATCATC[ATGC]{58}TGTTGC
    l    :length of read
    Q1   :threshold for low quality score [20].
    Q2   :maximum percent of low-quality bases[0.2].
    h    :display the help information.
EOQ
exit(0);
}
```
